# BioMeta: modular reprogrammable metasurface for noninvasive human respiration monitoring

**DOI:** 10.1515/nanoph-2025-0050

**Published:** 2025-03-27

**Authors:** Xin Yu Li, Long Chen, Shi Long Qin, Ke Zhan Zhao, Zi Xuan Cai, Qiao Cong Peng, Qian Ma, Jian Wei You, Tie Jun Cui

**Affiliations:** State Key Laboratory of Millimeter Wave, Southeast University, Nanjing 210096, China

**Keywords:** modular reprogrammable metasurface, metasurface-based sensing, human respiration monitoring, noise suppression, motion-robust sensing

## Abstract

Human vital-sign sensing using electromagnetic wave has emerged as a promising technology for the noninvasive monitoring of individuals’ health status. Here, a modular reprogrammable metasurface system is presented to suppress noise in noninvasive human respiration sensing. The proposed reprogrammable Biological Metasurface (*BioMeta*) provides three-dimensional dynamic control over wavefront shaping and thus can reduce interference from human limb motions. This capability allows the system to acquire health data accurately and reliably and is particularly beneficial in real-world environments where human subjects may change posture or location frequently. Furthermore, the meta-atom in *BioMeta* is modular and detachable, thereby resulting in reusable properties and promoting environmental sustainability. Meanwhile, the characteristics of mechanical control enable *BioMeta* to operate without continuous power supply, thus saving energy to a certain extent. A contactless human respiration sensing prototype based on the proposed *BioMeta* is demonstrated. Experimental results validate that the *BioMeta* system can accurately monitor the breathing of multiple individuals with limb movements by means of time multiplexing, with an average estimation error of 0.5 respiration per minute. The proposed system enhances sensing accuracy and reliability for noninvasive human respiration monitoring, presenting a versatile and environmentally friendly solution for applications like elderly care and disease monitoring.

## 1 Introduction

Metamaterials and metasurfaces have garnered substantial attention in both academic and practical domains due to their unprecedented capability of controlling electromagnetic (EM) waves [1], [2], [3], [4], [5]. Among them, information metamaterials enable to adjust EM parameters in a digital and programmable manner, opening up vast opportunities in fields that demand precise EM wave manipulation, such as wireless communications, the Internet of Things, and biomedical engineering [6], [7], [8], [9], [10], [11], [12], [13]. In particular, the information metamaterials have significantly enhanced the adaptability and functionality of EM wave-based human vital-sign sensing systems, which can revolutionize the way of detecting human physiological movements associated with vital signs [14], [15], [16], [17]. Compared to the traditional vital-sign monitoring approaches relying on wearable and implantable sensors, the EM-based contactless sensing technology is capable of monitoring the vital signs continuously without physical contact, offering a compelling alternative for healthcare, remote patient monitoring, and noninvasive diagnostics [18], [19], [20], [21]. Moreover, the nonintrusive nature of this sensing strategy is advantageous in situations where physical contact is infeasible, such as in infectious disease wards or during sleep studies [22], [23], [24], [25], [26].

Despite of remarkable progress, the existing noninvasive vital-sign sensing systems are susceptible to interference from body movements, hindering their applications to vital-sign sensing for moving individuals [27], [28]. Specifically, when the human individual is moving (e.g., walking and waving), the reflected signals from limb motions of the individual may be entangled with or even overwhelm the chest echo [29], [30], [31]. However, the limited spatial resolution of these systems prevents the separation of chest and limb echoes in the spatial domain [32], [33]. Meanwhile, there are similar harmonic frequency components in the chest and limb echoes, leading to the difficulty of distinguishing the chest echo from the limb echo in the frequency domain [34], [35], [36], [37]. For instance, Wang et al. [13] employed a 9.7 GHz metasurface to focus EM waves on the human chest and detect his vital signs (i.e., respiration and heartbeat) in real time. Furthermore, Li et al. [14] proposed a multiperson detection and vital-sign sensing system using the spatial-time-coding (STC) reprogrammable metasurface. With the adopted STC metasurface, the transmitted sensing signal was modulated into different harmonic beams for multiperson sensing. Although there have been some studies on vital-sign monitoring using metasurfaces [13], [14], [15], these works mainly focus on the perception of individuals in a stationary state without limb motions (e.g., sitting and standing *in situ*). Furthermore, these systems primarily utilize electrically controlled metasurfaces, without considering issues related to continuous power supply and fabrication costs.

Metasurfaces composed of electrical meta-atoms are faced with problems such as energy consumption [38], [39], [40], [41], [42]. Specifically, the PIN diode-based meta-atoms generally have limited degree of freedom, leading to a roughly discontinuous phase distribution that unavoidably introduces diffraction losses [43]. Meanwhile, though varactor diodes can provide larger phase levels, the distortion caused by amplitude-phase correlation often occurs [44]. Moreover, each electrical meta-atom requires at least one PIN diode or varactor diode, which requires a continuous power supply about several hundred milliwatts and thus leads to a trade-off between the energy consumption and metasurface size [45], [46], [47]. Even more, the electronically reprogrammable mechanism imposes more stringent requirements on the deployment of metasurfaces, hindering large-scale applications. To deal with these challenges, other alternative physical mechanisms have been proposed, which enable the reprogrammable functions without consuming electricity [48], [49], [50].

**Figure 1:**
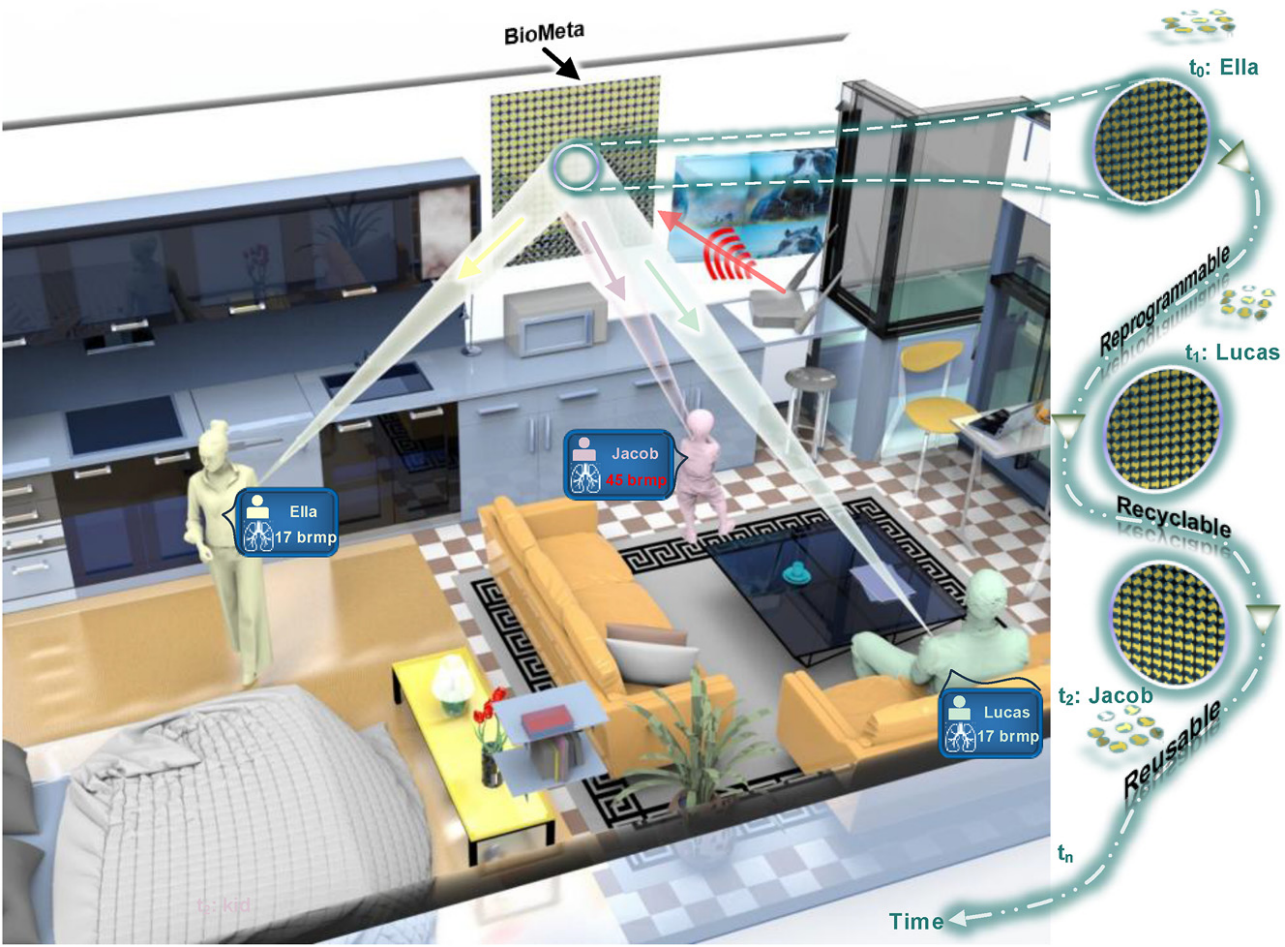
Schematic of the proposed *BioMeta* system for motion-robust human vital-sign sensing. The system is able to monitor the vital signs of both stationary and moving persons without contact. The programmable *BioMeta* is able to dynamically manipulate EM waves in 3D space, thus achieving physical-layer body noise suppression and ultimately leading to more accurate and reliable health data acquisition. Furthermore, the mechanically controlled *BioMeta* only requires power feeding during codebook switching, making it more suitable for long-term and low-power health monitoring. The detachable meta-atoms are recyclable and can be attached to diverse furniture surfaces in arbitrary shapes, exhibiting significant potential for applications in environmentally friendly indoor healthcare scenarios.

Here, we propose the Biological Metasurface (*BioMeta*), which is a reprogrammable and detachable metasurface platform to suppress noise in noninvasive human respiration sensing, as illustrated in Figure 1. By mechanically rotating each meta-atom, the *BioMeta* could achieve continuous and accurate phase control and thus focus EM waves at a specific location in the three-dimensional (3D) space. As a result, the proposed system can effectively reduce disturbances from random body movements and enhance the quality of physiological signals, offering significant potential for high-performance health monitoring. Furthermore, compared to electrically controlled metasurfaces, the proposed *BioMeta* does not require continuous power supply after codebook switching, making it more energy-efficient for long-term and low-power elderly care. Moreover, due to the modular characteristics, *BioMeta* can be attached to diverse furniture surfaces in arbitrary shapes, exhibiting significant potential for applications in smart home systems. Besides, the detachable meta-atom in the *BioMeta* is recyclable and reusable, which can reduce fabricating cost and thus is environmentally friendly. A contactless human respiration sensing platform using the proposed *BioMeta* is built. Experimental results demonstrate that the *BioMeta* system can accurately monitor the breathing of multiple individuals with limb movements by means of time multiplexing, with an average estimation error of 0.5 respiration per minute (RPM). The proposed system offers enhanced accuracy and reliability in health data acquisition, presenting a versatile and power-efficient solution for applications like elderly care and disease monitoring.

## 2 Results and discussion

**Figure 2:**
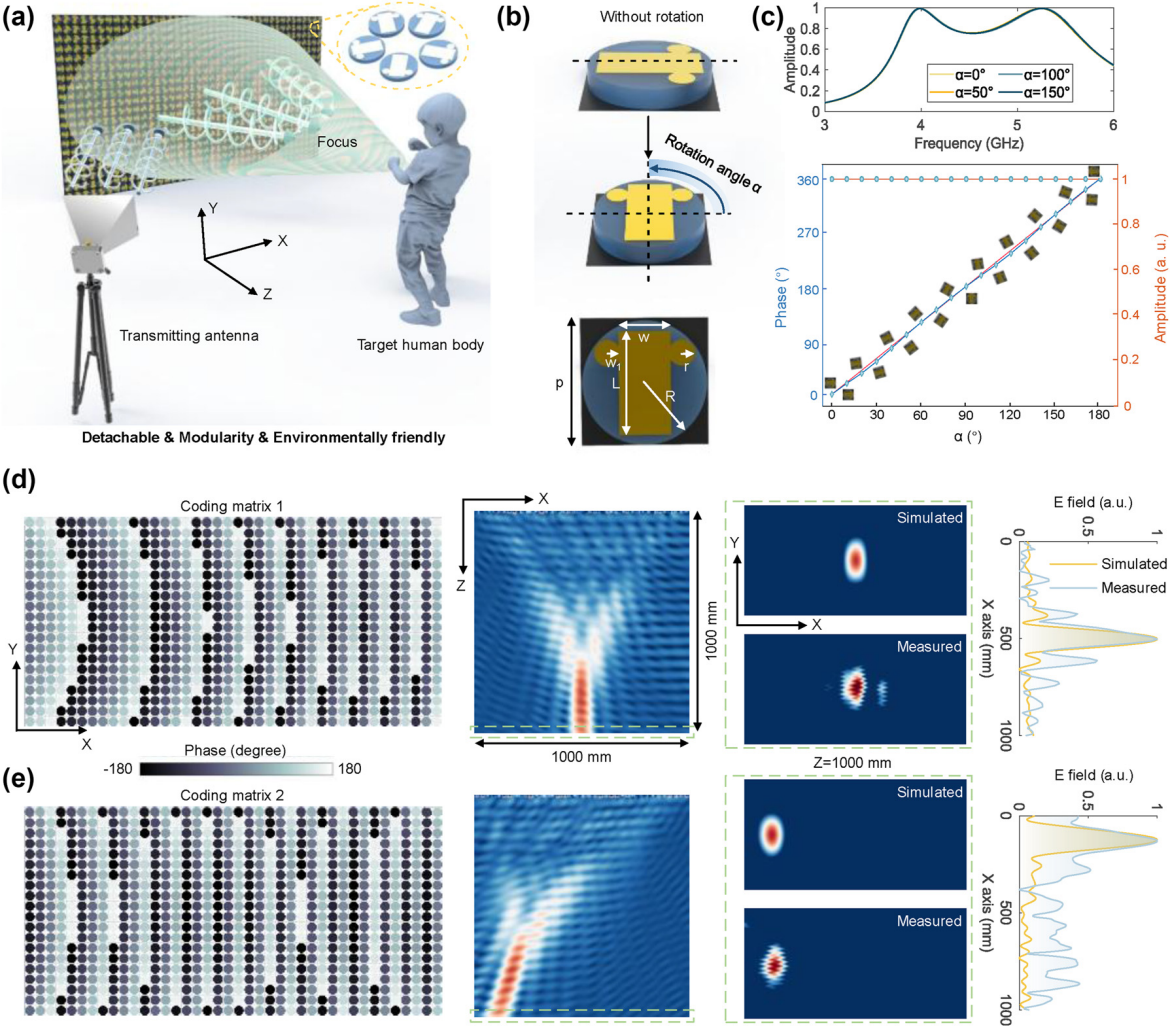
The proposed programmable *BioMeta*. (a) The *BioMeta*-based EM wave focusing. The modular and detachable meta-atom can be attached on anywhere in the room for EM wave manipulation, endowing the proposed system with more freedom of applications. (b) Programmable and modular meta-atom, which can be rotated using a rotary motor. There are 19 rotation angles *a*, allowing the proposed *BioMeta* to precisely control 360° phase of EM waves. (c) The amplitude and phase properties of the proposed *BioMeta.* It is observed that the phase of EM waves can be changed by different rotation angles ranging from 0° to 180°. (d), (e) Different coding matrices of the *BioMeta* (left), the resultant electric field distributions on the *xoz* (left-of-center) and the *xoy* planes (right-of-center), and the normalized electric field (right). The programmable characteristics enable *BioMeta* to focus EM waves on different locations, thus achieving vital-sign sensing of multiple persons.

We propose the modular and reprogrammable *BioMeta* to reduce disturbances from random body movements in noninvasive human respiration sensing, as illustrated in Figure 2. As illustrated in Figure 2(a), when utilizing the proposed *BioMeta* in contactless human vital-sign sensing, EM waves can be focused on specific 3D regions of interest, such as the human chest. As a result, the interference from human limbs and other environmental interference can be significantly reduced, remarkably enhancing the signal-to-noise ratio (SNR) of human physiological signals and improving the accuracy and reliability of vital-sign measurements. To achieve this, the modular and mechanically controlled meta-atom is designed, which can be rotated across 19 distinct rotating angles a achieving 360-degree granular control over the phase of EM waves. The rotational characteristics of the metasurface are illustrated in Figure 2(b), with the rotation angle denoted as *α*. It could be observed that the proposed *BioMeta* is a typical Pancharatnam–Berry (PB) phase reflective metasurface, renowned for its high phase manipulation capabilities. The entire unit cell is arranged in a metallic square lattice with a period of *p* = 25 mm, and the radius of the circular substrate is *R* = 12 mm. The dimensions of the rectangular strip are *L* = 20 mm and *w* = 10 mm, the radius of the circular elements is *r* = 2.5 mm, and the distance between the center of the circular elements and the edge of the rectangular strip is *w*_1_ = 2.14 mm. In this case, the phase of the transmitted EM waves can be finely tuned to meet the desired requirements of human vital-sign sensing by varying the rotation angles of the meta-atoms.

Compared to electrically controlled metasurfaces, the mechanically controlled *BioMeta* does not require continuous power supply after finishing codebook switching, making it more suitable for long-term monitoring, especially in low-power environments like smart homes or healthcare facilities. For instance, we assume that the metasurface employed for vital-sign sensing consists of 800 meta-atom units. There are 4 PIN diodes in each electrically controlled meta-atom to achieve 16 types of phase control, with each PIN diode consuming approximately 0.7 mW. Then, the electrically controlled metasurface will consume 44.8 J of energy when collecting the physiological signals for 20-s respiration monitoring. In contrast, the proposed *BioMeta*’s energy consumption for physiological data acquisition is almost 0 in this duration. Meanwhile, in terms of manufacturing cost, each *BioMeta* unit costs approximately 9 RMB, while each PIN diode costs around 10 RMB. Compared to the electrical meta-atom, the *BioMeta* unit offers a cost reduction of 77.5 %. Furthermore, the modular meta-atom is detachable and recyclable, which not only reduce costs but also promote environmental sustainability. They can be attached to various surfaces in the indoor environment, providing increased degree of flexibility in application and deployment.

The amplitude and phase variations to rotation angle of the meta-atom are provided in Figure 2(c). It could be observed from these figures that the proposed metasurface provides dynamic control over wavefront shaping, enabling the system to adjust in real time for varying distances, body positions, and movements. Such flexibility is particularly beneficial in real-world environments where human subjects may change posture or location frequently. Figure 2(d) and (e) illustrates the coding matrices of the *BioMeta* (left) as well as the resultant electric field distributions on the *xoz* (middle) and *xoy* planes (right) when focusing EM waves on two different locations, highlighting the system’s ability to control EM wave propagation in three-dimensional space. The reprogrammable characteristic enables the *BioMeta* to dynamically manipulate EM waves, allowing for multiple individuals to be monitored simultaneously, or enabling large-area scanning for group health monitoring. These combined features make the *BioMeta* a powerful tool to achieve dynamic, precise, energy-efficient and environmentally friendly health monitoring.

**Figure 3:**
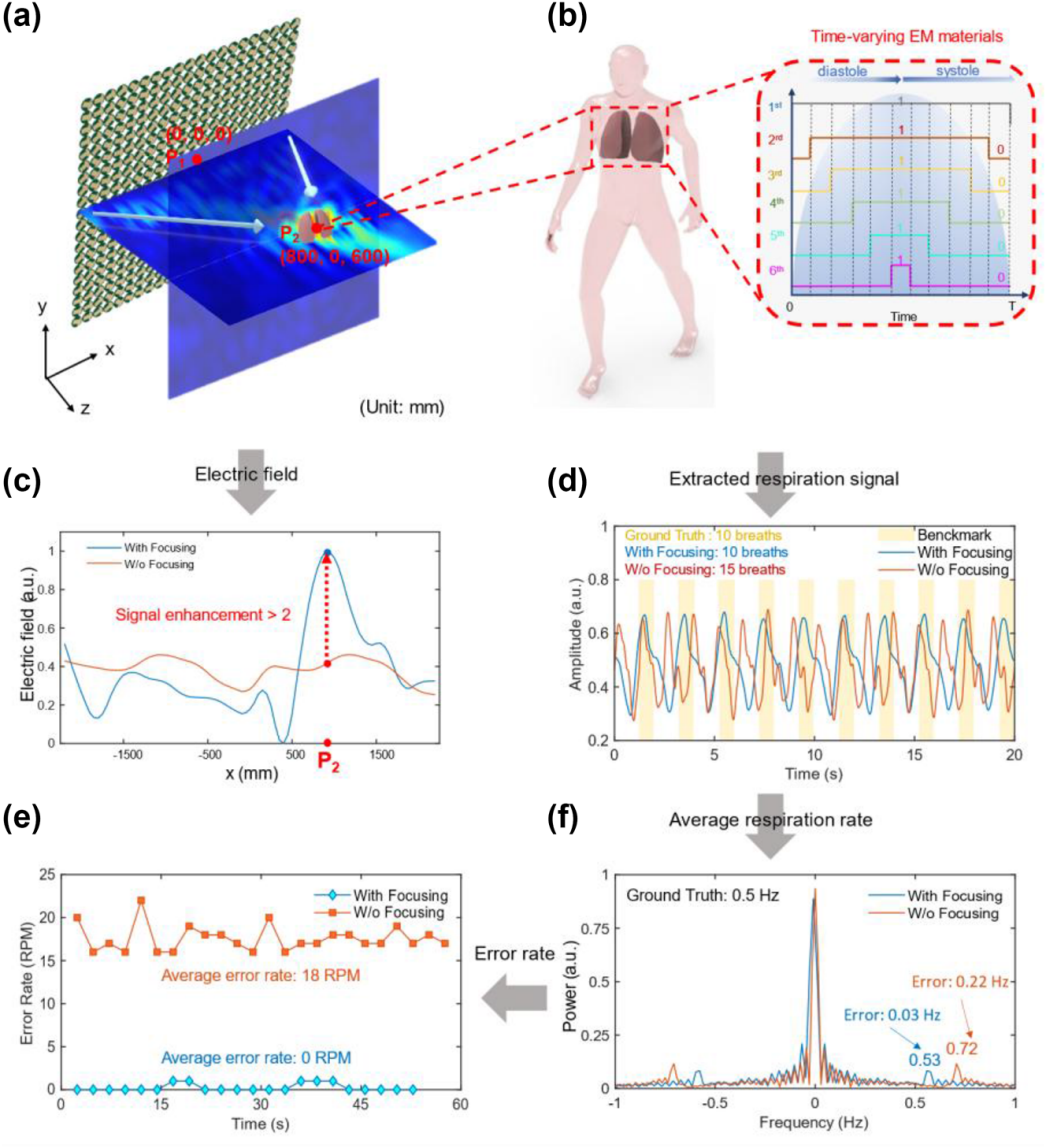
Simulation of *BioMeta*-based vital-sign sensing for moving persons. (a) EM wave focusing empowered by the *BioMeta*. The Gerchberg–Saxton (GS) algorithm is employed to optimize the coding of the metasurface. (b) The construction of human body model with limbs movements and respiration. The limbs and the chest of the human model are simulated with customized time-varying EM materials. (c) The intensity of EM waves at diverse locations with and without (w/o) metasurface-based focusing. The *x*-axis coordinate is variable in this case, and the *y*-axis and *z*-axis coordinates are fixed at 0 and 600 mm, respectively. It could be observed that with *BioMeta*-based focusing, most energy of the EM wave is concentrated on the chest. (d) The extracted human respiration signals in the two scenarios. It is noted that the benchmark signal is utilized as the ground-truth of respiration, and preset before the simulation. Each peak in the signal represents one breath taken by the human model. (e) The error rate of respiration estimation with and w/o metasurface-based focusing. The average error rate of respiration estimation with EM wave focusing is approximately 0 RPM. (f) The average respiration frequency in the two scenarios.

We further conduct some simulations to demonstrate the feasibility of the proposed *BioMeta* system for human vital-sign sensing. The simulated results are illustrated in Figure 3. As illustrated in Figure 3(a), the proposed mechanically reprogrammable metasurface is modeled for EM wave manipulation. A plane wave is utilized as the excitation of the metasurface. The Gerchberg–Saxton (GS) algorithm (see Note S1, Supplementary Information for more details) is employed to optimize the code patterns of the reprogrammable metasurface for EM wave focusing. In this way, most of the EM waves are concentrated on the individual’s chest, significantly improving the power of human chest echoes, as illustrated in Figure 3(c). Compared to the case without focusing (marked in red line) in Figure 3(c), the metasurface-based focusing (marked in blue line) is capable of enhancing the intensity of EM waves at the chest position while reducing the intensity at surrounding locations. Furthermore, by redirecting EM waves toward the individual’s chest, reflections from the surroundings and human limbs could be minimized, endowing the system to eliminate the environmental interference and body noise without sophisticated echo signal processing.

During the simulation, we propose a time-varying human EM model composed of two customized time-varying EM materials to simulate the human respiration and the movements of human limbs, respectively. Specifically, the normalized conductivity of different layers in the EM human lung model during a cycle of respiratory movement is shown in Figure 3(b). As the electrical conductivity of each layer changes periodically, the periodic movements of the lungs can be simulated. It is noted that the parameters of the time-varying material, such as the number of layers, periodicity, and conductivity, are adjustable based on specific requirements. Although we do not provide additional simulated results in the article, we have varied these parameters and conducted repeated experiments for verification. Using the dynamic EM human model, the simulated human physiological signals under two scenarios, i.e., the scenarios with and w/o metasurface-based focusing, are obtained, as are shown in Figure 3(d)–(f). The theoretical analysis of time-varying systems and the simulated human echo signals arrived at the receiver are provided in Note S2 and S3, Supplementary Information, respectively. Then, the respiration signals under the two scenarios are extracted from the simulated human echo signals, which are illustrated in Figure 3(d). We can observe that the metasurface-based focusing can effectively eliminate the interference of limb motions and thus produce high-quality human chest echo signal for vital-sign sensing (marked in blue line). In contrast, when the EM wave is not focused (marked in red line), the limb echo and chest echo are entangled with each other, hindering accurate extraction of respiration signal. The estimated respiration frequency and estimation error rate are illustrated in Figure 3(e) and (f). It is indicated that the proposed metasurface-based approach can accurately estimate the respiration rate of the human model, with an average error of 0.03 respirations per minute. The simulations demonstrate that the proposed *BioMeta* can enhance the energy of vital-sign signal while effectively suppressing body noise, thereby achieving motion-robust human vital-sign sensing.

**Figure 4:**
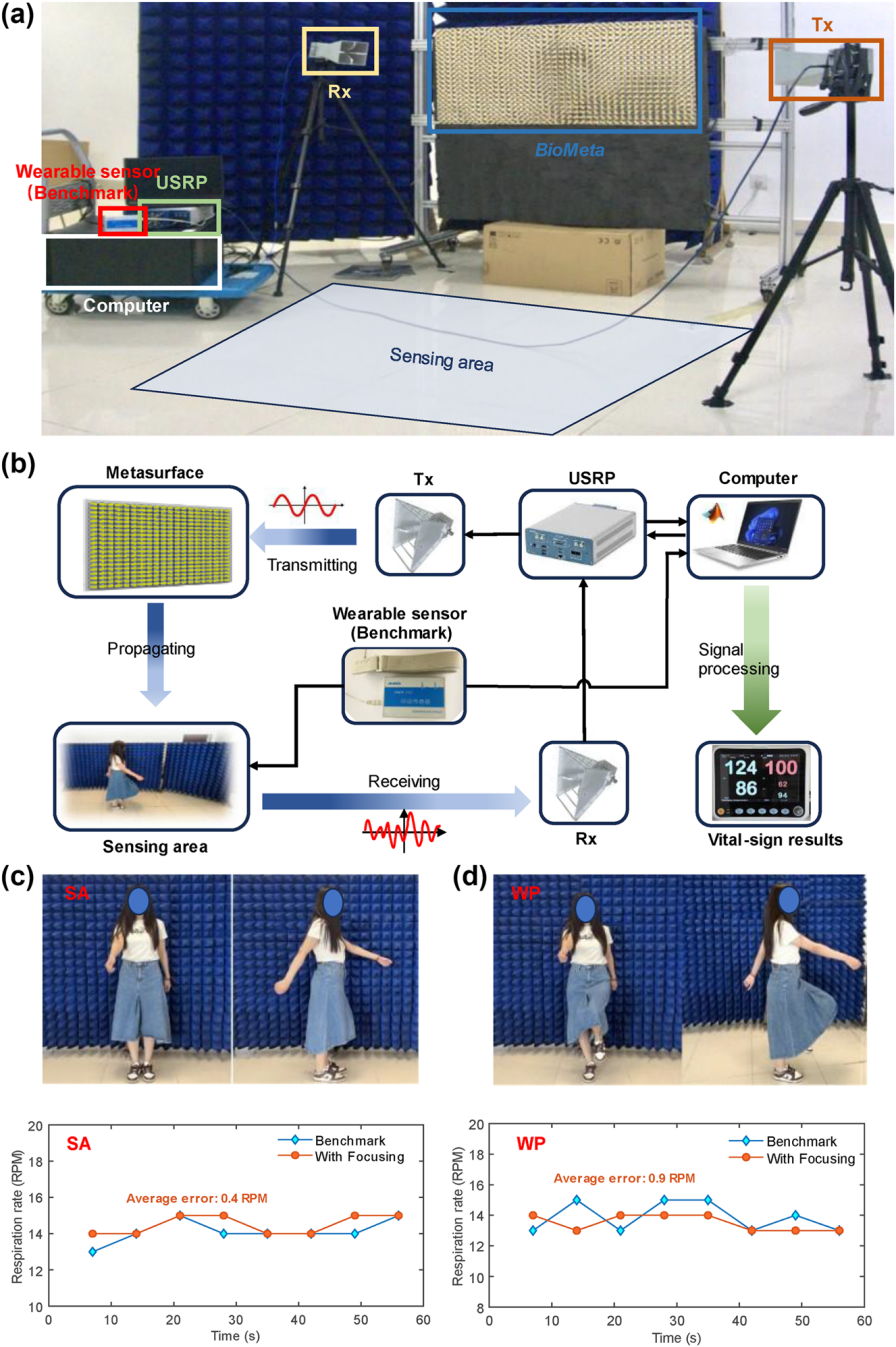
Experimental setup. (a) Experimental platform with the proposed *BioMeta*, a Universal Software Radio Peripheral (USRP) device, a computer, a benchmark wearable sensor, and two horn antennas for signal transmitter and receiver. (b) Schematic of the experimental deployment and connection. (c) Experimental results of the proposed *BioMeta* for single-person respiration sensing with arm swinging. (d) Experimental results for single-person respiration sensing with walking in place.

We also conduct some experiments to verify the effectiveness of the proposed *BioMeta* system for motion-robust human vital-sign sensing. The experimental setup is displayed in Figure 4 and Table 1. As shown in this figure, the experimental platform is composed of the proposed low-cost and low-power mechanically reprogrammable metasurface, a Universal Software Radio Peripheral (USRP) device, a computer, a benchmark wearable sensor, and two horn antennas. The USRP device USRP-2974 is utilized as the sensing signal generator. Meanwhile, two high-gain horn antennas fixed on the tripods at a height of 1.3 m are employed for transmitting and receiving signals, respectively. The computer is connected to the USRP and the wearable sensor for data processing and time synchronization. The USRP transmits EM waves to the metasurface and then receives the signals carrying a wealth of environmental information, including human vital-signs. The wearable sensor is adopted to collect abdominal pressure signal, which is an effective representation of human respiration movements and can be utilized to obtain the ground-truth of respiration rate. It is noted that when the *BioMeta* system is utilized in practice, the wearable sensor does not need to be worn. The experiments were conducted in an indoor laboratory environment with multiple tables and chairs, as well as some persons passing by. Then, we first conduct experiments of respiration sensing for a single person using the experimental platform in Figure 4(a). The estimation results of the person’s respiration rate under different moving patterns, including swinging arms (SA) and walking in place (WP), are illustrated in Figure 4(c) and (d). We can find that the average error in estimating a walking person’s respiration rate is larger than that of a person waving her arms. Specifically, when utilizing the proposed *BioMeta* for EM wave focusing, the average error in sensing the breath of the swinging individual is 0.4 RPM, 0.5 RPM lower than that when estimating the respiration rate of the walking individual.

**Table 1:** Experimental parameter setting.

Parameters	Experimental setting
Center frequency	3.9 GHz
Sampling rate	100 kHz
Antenna gain	11.1 dBi
Environment temperature	∼20 °C

**Figure 5:**
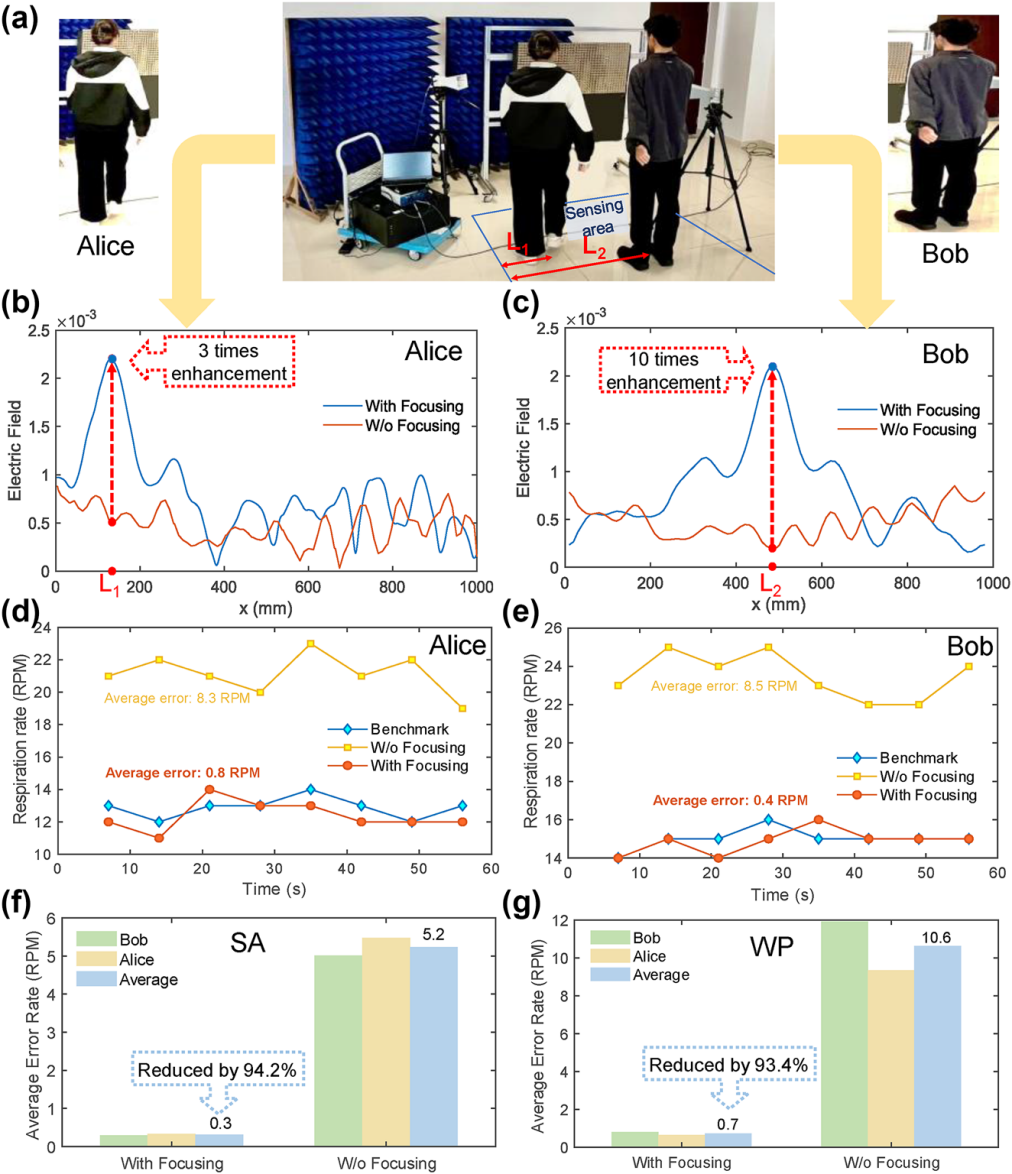
Experimental results of the proposed *BioMeta* for motion-robust multiperson respiration sensing. (a) The experimental scenarios where the human individuals *Alice* and *Bob* are at the location *L*_*1*_ and *L*_*2*_ for sensing, respectively. *Alice* is walking in place, while *Bob* is swinging his arms. (b), (c) The measured electric field distributions when perceiving *Alice*’s and *Bob*’s vital signs, respectively. (d), (e) The estimated results of respiration rate for *Bob* and *Alice*, respectively. (f) The average error of estimating respiration rate when the individuals are swing their arms. (g) The average error of estimating respiration rate when the individuals are walking in place.

Meanwhile, the experimental results of the proposed *BioMeta* for motion-robust multiperson respiration sensing are illustrated in Figure 5. Experimental results demonstrate that the *BioMeta* system can accurately monitor the breathing of multiple individuals, even with limb movements, through time multiplexing. Specifically, there are two persons (i.e., Bob and Alice) at different locations to be perceived their vital signs, as shown in Figure 5(a). It could be calculated that the system can distinguish between two individuals close to each other with an angular resolution of about 11°. During the experiments, both Bob and Alice move in two manners, which are swinging his/her arms and walking in place. To accurately estimate the respiration rate of the two individuals, the proposed reprogrammable metasurface sequentially adjusts its code patterns according to the chest positions of two individuals and focuses the EM waves on each individual’s chest. The details of oblique focusing are provided in Note S4, Supplementary Information. Furthermore, to prove the effectiveness of the proposed method, we replace the metasurface with a metal surface of equal size and collect physiological signals of moving individuals without EM wave focusing for comparison. Despite its passive nature and reflection losses, a widely used metal plate serves as a conventional reflective surface in practical scenarios. Therefore, we use the metal plate as a comparison to prove the effectiveness of the proposed metasurface-based EM wave focusing method for noise suppression. The configuration of the comparison experiment is provided in Note S5, Supplementary Information. It is noted that all other configurations are the same in the comparison experiment, except for the difference in the EM reflection medium. Then, the electric field distributions of the metal surface and the *BioMeta* are illustrated in Figure S8(c) and (d), respectively. It could be observed that the *BioMeta* is capable of concentrating the energy of the EM wave at a specified location, while the electric field distribution of the EM wave reflected by the metal plate is random. As a result, there is less noise in the physiological signal extracted in the case of focusing than that w/o focusing. Then, Figure 5(b) and (c) illustrates the comparison results of EM wave intensity around the chest of Bob and Alice when there is a metasurface (With Focusing) and a metal surface (W/o Focusing) for EM wave manipulation, respectively. It could be observed that the proposed BioMeta system could improve the power of the EM waves around the individual’s chest, while reducing the energy of the EM waves reaching other locations. In this way, the echo signals reflected by the moving limbs, which are considered as noise in the human vital-sign sensing task, can be significantly weakened, thereby improving the SNR of physiological signals and achieving motion-robust vital-sign perception. Figure 5(d) and (e) provides the respiration rate estimations of the proposed system when estimation Bob and Alice’s respiration rate within 60 s. We can find that the proposed system has achieved the estimated respiration rate, which is close to the benchmark, with average error of 0.4 RPM and 0.8 RPM, respectively.

Moreover, as shown in Figure 5(f) and (g), compared to traditional algorithm without EM wave focusing, the proposed algorithm reduces respiration estimation error by over ten times when there are macro movements in human limbs. Besides, when there are macroscopic human body motions, the proposed system could significantly improve the accuracy of respiration rate estimation by focusing EM wave on the human chest (see Note S6, Supporting Information for more details). Meanwhile, we also compare the performance of the proposed system for respiration monitoring when the individuals are swinging their arms and walking in place, as shown in Figure 5(f) and (g). Specifically, the average error of estimating respiration rate when the individuals are swinging their arms and walking in place is 0.3 RPM and 0.7 RPM, respectively, which is significantly lower than the error w/o EM wave focusing. It is mainly due to that when the person is walking in place, there are more body noise induced by the legs, resulting in interference on the human chest echo. In a word, the experimental results in Figure 5 demonstrate that the *BioMeta* can improve the power of the EM wave reaching the human chest by focusing EM waves on the specific location, thus enhancing the SNR of the chest echo. As a consequence, the human body noise induced by limb motions could be remarkably mitigated, resulting in high-quality physiological signals and accurate vital-sign monitoring.

When conducting experiments in a real environment, the proposed *BioMeta* system focuses most of the EM waves on the human thoracic region, minimizing the impact of background environment on the human echo signal. Additionally, static background clutter can be removed through signal processing methods, ensuring that the overall performance does not degrade significantly. Moreover, the experimental results of respiration rate estimation for different individuals are provided in Figure S9, Supporting Information. It can be observed that the performance of BioMeta remains stable when it is used for sensing the vital signs of different individuals, with an average estimation error of approximately 0.5 RPM. In future studies, we will evaluate the proposed method across various scenarios and individuals to further validate its effectiveness.

## 3 Conclusions

We proposed the modular and reprogrammable *BioMeta* system, which significantly advanced noninvasive human respiration monitoring by offering enhanced accuracy and reliability. Its ability to suppress interference from limb motions, coupled with its modular, detachable design, ensured reusability and environmental sustainability. The system’s low energy consumption and potential for long-term operation without a continuous power supply further enhanced its practicality. Experimental results demonstrated the system’s capability to monitor respiration of the individuals with limb motions, with an average respiration estimation error of 0.5 RPM. Compared to the conventional algorithm without EM wave focusing, the proposed system improved the respiration sensing performance by more than ten times when there are macro-level movements in the human limbs. Furthermore, the reprogrammable property enabled the *BioMeta* system to accurately monitor the breathing of multiple individuals with limb movements. The modular, environmentally friendly and motion-robust characteristics of the *BioMeta* system highlight its potential for large-scale deployment in smart homes and healthcare settings, offering a promising alternative to traditional wearable and implantable sensors.

## 4 Methods

### 4.1 Experimental setup and configuration details

In the experiment, the software LABVIEW-2019 from National Instruments (NI) was adopted to control the whole vital-sign sensing system. Specifically, a LABVIEW project was created in which the baseband sensing signal was first generated with the LABVIEW code and subsequently sent to the air by the USRP. Then, the echo signal processing module was implemented with MATLAB script and integrated into the LABVIEW project. Serial port communication between the computer and the wearable sensor was also realized with LABVIEW for data transmission. Meanwhile, since the benchmark signal collected by the wearable sensor was utilized to verify the performance of the proposed *BioMeta* system, timestamp-based synchronization between the benchmark signal and the human echo signal was indispensable. Additionally, since human respiration rate was estimated based on the periodic property of physiological movements, data accumulation over a long period was necessary to obtain sufficient frequency information of respiratory movements. Therefore, the duration of each physiological signal was 15 s.

### 4.2 Human echo signal processing

Human respiration sensing involves the detection and analysis of human physiological signal, which are often embedded within complex and noisy environments. Therefore, an effective signal processing algorithm is crucial to extract physiological signals from human echoes and achieve accurate respiration sensing. In this article, we adopted the improved Variational Mode Decomposition (VMD) algorithm [14] for human echo signal processing, which is a cutting-edge signal processing technique that has gained significant attention for its robust ability to decompose complex signals into their intrinsic mode functions (IMF). Unlike traditional methods such as Empirical Mode Decomposition (EMD) or wavelet transforms, VMD offers enhanced stability, adaptability, and precision, making it particularly suitable for applications in human vital-sign sensing.

In the improved VMD algorithm, a frequency-correlated penalty factor *α* was proposed for adaptively extracting IMFs with narrower bands. Specifically, for the *i*th IMF (*i* ≤ *I*), its penalty factor *α*_*i*_ can be denoted as

(1) $$\alpha_{i}=\alpha_{\text{int}}\left(-\zeta {\left\|\omega_{i}-\omega_{r}\right\|}_{2}^{2}\right)$$

where *α*_int_ denotes the initial penalty factor, and *ζ* is the scale factor. *ω*_*i*_ is the frequency of the ith IMF, and *ω*_*r*_ denotes the reference frequency and is preset based on the observation that the respiration frequency. With the adaptive *α*_*i*_, a greater penalty will be assigned when the center frequency of the *i*th IMF is approaching its reference *ω*_*r*_, resulting in a narrower-band signal. The whole human echo signal processing pipeline was performed in MATLAB. In the experiments, we first filtered the out-of-band noise in the human echo signal and then employed the improved VMD algorithm to extract the respiration signal. At last, peak detection was utilized to estimate human respiration rate.

## Supplementary Material

Supplementary Material Details
