# Supplementary material for: BioMeta: modular reprogrammable metasurface for noninvasive human respiration monitoring
Source: Nanophotonics. 2025 Mar 27;14(7):981–91. doi: 10.1515/nanoph-2025-0050 (PMC11980868; doi:10.1515/nanoph-2025-0050)
Supplement: Supplementary file 1 — Supplementary Material Details [file j_nanoph-2025-0050_suppl_001.pdf]

Supplementary Material for: "BioMeta: Modular Reprogrammable Metasurface for Non-Invasive Human Respiration Monitoring"

Xin Yu Li[a], Long Chen[a], Shi Long Qin[a], Ke Zhan Zhao[a], Zi Xuan Cai[a], Qiao Cong Peng[a], Qian Ma[a], Jian Wei You[a]*, and Tie Jun Cui[a]*

[a]Southeast University, School of Information Science and Engineering, State Key Laboratory of Millimeter Wave, Nanjing, China

*Jian Wei You, jvyou@seu.edu.cn; *Tie Jun Cui, tjcui@seu.edu.cn;

1 Focusing Algorithms for the *BioMeta*

Common approaches to achieve focusing are typically based on numerical computational formulas. Since focusing can be regarded as a subset of imaging problems, in this section, we introduce the classical imaging algorithm we employed, namely the Gerchberg-Saxton (GS) algorithm. The GS algorithm is an iterative phase retrieval algorithm that recovers phase information from intensity measurements. It was introduced by Roy Gerchberg and Warren Saxton in 1972. By acquiring intensity information on two different planes (such as the incident optical field plane and the imaging plane, or the image plane and the far-field Fourier plane, with the latter often being the preferred choice), known and constrained conditions are used to iteratively recover the wavefront phase through forward and backward propagation, thereby obtaining complex amplitude image information. The classical GS algorithm relies on Fourier transforms and their inverses to calculate the propagation of the optical field between two planes, based on the principle of angular spectrum propagation.

The computational process of the GS algorithm is illustrated in Fig S1 and includes the following steps: (1) Estimate or randomly generate the initial phase distribution. (2) Use the incident plane amplitude information and combine it with the phase distribution to obtain the complex amplitude information of the incident plane. (3) Forward propagate the complex amplitude information from the incident plane to the output plane to obtain the complex amplitude information at the output plane. (4) Use the known amplitude information at the output plane as a constraint to replace the real amplitude component in the propagated complex amplitude distribution. (5) Backward propagate the modified complex amplitude image to the incident plane to obtain a new complex amplitude image, and repeat steps (2)-(5) until convergence. The GS algorithm is an iterative computational process and is therefore a form of error reduction algorithm.

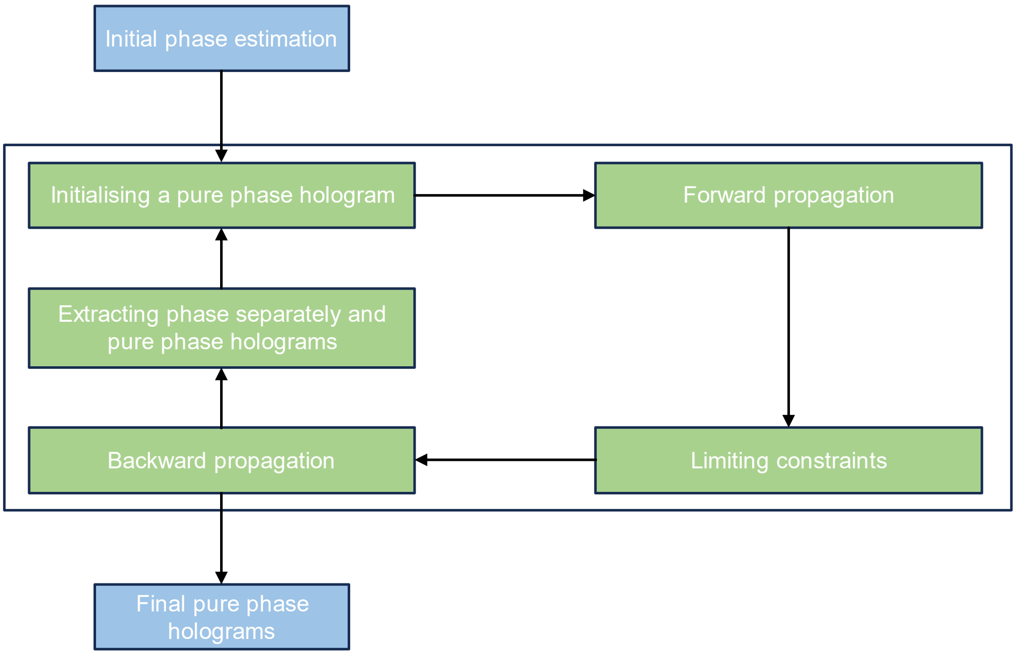

Fig S1 Flowchart of GS algorithm calculation.

```
A = IFT(Target)
while error criterion is not satisfied
B= Amplitude(Source) * exp(i*Phase(A))
C = FT(B)
D= Amplitude(Target)* exp(i*Phase(C))
A = IFT(D)
end while
Retrieved Phase = Phase(A)
```

Fig S2 GS algorithm pseudo-code.

The detailed steps are as follows:

Step1. Randomly set an initial phase $\varphi(x, y)$ on the input plane and combine it with the known amplitude distribution $|F(x, y)|$ to form the incident optical wave function $f(x, y)$.

Step2. Apply a Fourier transform to $f(x, y)$ to obtain the optical wave function $g(u, v)$ on the output plane.

Step3. Combine the phase of $g(u, v)$ with the amplitude distribution $|G(u, v)|$ on the output plane to form the function $g'(u, v)$.

Step4. Apply an inverse Fourier transform to $g'(u, v)$ to obtain the next iterative wave function $f'(x, y)$.

Step5. Substitute the phase part of $f'(x, y)$ into Step 1, replacing $\varphi(x, y)$. Repeat the above steps until the mean square error (MSE) and Sum-Square Error (SSE) are less than the specified threshold ε:

$$SSE = \left[\iint (g(u,v) - |G(u,v)|)^2 dudv\right] / [|G(u,v)|^2 dudv] < \varepsilon$$

The pseudocode is shown in Fig S2. The GS algorithm serves as the foundation for many iterative computational phase imaging algorithms and finds applications in fields such as coded imaging, tomographic imaging, and holographic imaging. Despite its ability to simply retrieve phase distribution information, the GS algorithm may converge to local minima due to its inherent limitations. To address this issue, derivative algorithms of the GS algorithm have been developed, such as the weighted Gerchberg-Saxton algorithm, adaptive algorithms, the Fienup algorithm, and the Hybrid Input-Output (HIO) algorithm. These methods improve the phase retrieval process by introducing new iterative strategies, optimizing error functions, and incorporating weighting functions during the update of complex amplitudes.

2 Analysis Theory of Time-Varying System

With the rapid advancement of science over the past two centuries, the role of time has emerged as a crucial factor in unravelling countless mysteries of the physical world. However, most fundamental physics assumes that physical systems are passive, merely responding to external stimuli. In explaining certain multiphysics or nonlinear phenomena, we have the opportunity to extend our understanding of wave phenomena in temporally non-uniform systems. Nevertheless, substantive progress in this field has been hindered by a lack of extensive research. Concurrently, the rise of metamaterials over the past two decades indicates that many fundamental aspects of wave physics under the guidance of classical theories in electromagnetism, acoustics, and elasticity remain to be explored. Recently, the exploration of new forms of wave-matter interactions has sparked significant interest in time as a new degree of freedom. In this process, a fundamental understanding of time-domain wave phenomena is essential. This understanding is primarily theoretical, with some experimental aspects, achieved through explicit temporal modulation of physical system parameters. It is important to note that the aforementioned time-varying media fall under the category of dynamic electromagnetic (EM) problems. By introducing time as a new degree of freedom, the design process becomes more enriched, garnering widespread attention.

Thus far, researchers have made significant advances in developing numerical methods to solve static electromagnetic problems. However, the numerical simulation of dynamic electromagnetic problems remains a formidable challenge. To achieve full-wave numerical analysis for monitoring the life status of human bodies, this is undoubtedly a dynamic problem. Therefore, this section will introduce a numerical computation method to address time-varying media problems—the Time-Domain Finite Integration Technique (TDFIT).

In 1966, K.S. Yee introduced a sophisticated spatial discretization scheme. This grid strategically places electric field components along edges and magnetic field components at the cell centers. Such an arrangement ensures that each electric field component is surrounded by four magnetic field components and vice versa. This spatial configuration inherently satisfies Faraday's law of electromagnetic induction and Ampère's circuital law. Consequently, the Yee grid has been widely applied in time-domain algorithms and helped develop the renowned Finite-Difference Time-Domain (FDTD) method. The Yee grid is composed of hexahedral meshes. While this grid strongly adheres to Faraday's and Ampère's laws, its linear nature can lead to staircasing errors when approximating certain curved boundaries. To mitigate these errors, we propose the TDFIT method based on the Yee grid. Unlike the FDTD method, the TDFIT method is derived from the integral form of Maxwell's equations:

$$\oint_{\partial s} \boldsymbol{E} \cdot d\boldsymbol{l} = -\frac{\partial}{\partial t} \iint_{s} \boldsymbol{B} \cdot d\boldsymbol{s} \tag{S1}$$

$$\oint_{\partial \tilde{s}} \boldsymbol{H} \cdot d\boldsymbol{l} = \frac{\partial}{\partial t} \iint_{\tilde{s}} \boldsymbol{D} \cdot d\boldsymbol{s} + \iint_{\tilde{s}} \boldsymbol{J} \cdot d\boldsymbol{s} \tag{S2}$$

$$\oiint_{s} \boldsymbol{B} \cdot d\boldsymbol{s} = 0 \tag{S3}$$

$$\iint_{\tilde{s}} \boldsymbol{D} \cdot d\boldsymbol{s} = \iiint_{\tilde{v}} \rho_v \cdot dv \tag{S4}$$

where $\boldsymbol{E}$ and $\boldsymbol{B}$ represent the electric field strength and magnetic flux density, respectively. The surface $\boldsymbol{s}$ is defined as any three-dimensional (3D) surface on the primary cell, and ∂s represents its contour. Additionally, $\boldsymbol{H}$ and $\boldsymbol{D}$ correspond to magnetic field strength and electric displacement vector, respectively. $\boldsymbol{J}$ and $\tilde{s}$ denote current density and surface on the dual cells. Within the volume V, we consider various auxiliary cells, with charge density denoted by ρ_v.

For subsequent mathematical derivations, we decompose the closed path integrals into a series of line integrals over line segments. These line integrals can be accurately described by introducing a set of new variables, which will play a crucial role in the following analysis.

$$\begin{cases} \hat{e}_{k_i} = \int_{L_{k_i}} \boldsymbol{E} \cdot d\boldsymbol{l} \\ \hat{h}_{k_i} = \int_{L_{k_i}} \boldsymbol{H} \cdot d\boldsymbol{l} \end{cases} \tag{S5}$$

$$\begin{cases} \hat{\hat{d}}_k = \int_{\tilde{s}_k} \boldsymbol{D} \cdot d\boldsymbol{s} \\ \hat{\hat{b}}_k = \int_{s_k} \boldsymbol{B} \cdot d\boldsymbol{s} \\ \hat{\imath}_k = \int_{\tilde{s}_k} \boldsymbol{J} \cdot d\boldsymbol{s} \end{cases} \tag{S6}$$

where the symbols $\hat{\boldsymbol{e}}$ and $\widehat{\boldsymbol{h}}$ represent the voltages and magnetomotive forces along the edges of the primary and dual cells, respectively. The symbols $\hat{\hat{\boldsymbol{d}}}$, $\hat{\hat{\boldsymbol{b}}}$, and $\hat{\boldsymbol{\imath}}$ represent the electric flux, magnetic flux, and current density flux through the primary and dual cells, respectively. Substituting these parameters into Maxwell's equations, we obtain the corresponding spatially discrete forms.

$$\begin{cases} \boldsymbol{C}\hat{\boldsymbol{e}} = -\frac{\partial}{\partial t}\hat{\hat{\boldsymbol{b}}} \\ \widetilde{\boldsymbol{C}}\widehat{\boldsymbol{h}} = \frac{\partial}{\partial t}\hat{\hat{\boldsymbol{d}}} + \hat{\boldsymbol{\imath}} \end{cases} \tag{S7}$$

In the above equations, the symbols $\boldsymbol{C}$ and $\widetilde{\boldsymbol{C}}$ represent the discrete curl operators on the primary and dual cells, respectively. For example, the definition of the curl operator $\boldsymbol{C}$ can be expressed as follows:

$$\boldsymbol{C}\hat{\boldsymbol{e}} = \hat{e}_{x_{i,j,k}} + \hat{e}_{y_{i+1,j,k}} - \hat{e}_{x_{i,j+1,k}} - \hat{e}_{y_{i,j,k}} = -\frac{\partial}{\partial t}\hat{\hat{\boldsymbol{b}}}_{z_{i,j,k}} \tag{S8}$$

The subscripts (i, j, k) indicate the grid coordinates in a rectangular coordinate system. To facilitate the numerical solution of Maxwell's equations, it is necessary to introduce the integral forms of constitutive relationships, detailed below:

$$\begin{cases} \hat{\hat{\boldsymbol{d}}} = \boldsymbol{M}_{\varepsilon}\hat{\boldsymbol{e}} \\ \hat{\hat{\boldsymbol{b}}} = \boldsymbol{M}_{\mu}\widehat{\boldsymbol{h}} \\ \hat{\boldsymbol{\imath}}_e = \boldsymbol{M}_{\sigma}\hat{\boldsymbol{e}} \\ \hat{\boldsymbol{\imath}}_m = \boldsymbol{M}_{\kappa}\widehat{\boldsymbol{h}} \end{cases} \tag{S9}$$

where $\boldsymbol{M}_{\varepsilon}$ represents the average permittivity matrix, $\boldsymbol{M}_{\mu}$ represents the average permeability matrix, $\boldsymbol{M}_{\sigma}$ is the average conductivity matrix, and $\boldsymbol{M}_{\kappa}$ corresponds to the average effective magnetic loss matrix. In static electromagnetic field problems, these effective electromagnetic parameters are time-invariant constants. However, in dynamic electromagnetic field problems, these parameters vary with time. According to the Yee cell method, the diagonal form of these material parameter matrices can be defined as follows:

$$M_{\varepsilon_k}(t) = \frac{\hat{\hat{d}}_k}{\hat{e}_k} = \frac{D \cdot \tilde{s}_k}{\bar{\varepsilon}^{-1}(t) D \cdot l_k} = \bar{\varepsilon}(t)\frac{\tilde{s}_k}{l_k} \tag{S10}$$

$$M_{\mu_k}(t) = \frac{\hat{\hat{b}}_k}{\hat{h}_k} = \frac{\bar{\mu}(t)H \cdot s_k}{H \cdot \tilde{l}_k} = \bar{\mu}(t)\frac{s_k}{\tilde{l}_k} \tag{S11}$$

$$M_{\sigma_k}(t) = \frac{\hat{\hat{\imath}}_{e_k}}{\hat{e}_k} = \frac{J_e \cdot \tilde{s}_k}{\bar{\sigma}^{-1}(t)J_e \cdot l_k} = \bar{\sigma}(t)\frac{\tilde{s}_k}{l_k} \tag{S12}$$

$$M_{\kappa_k}(t) = \frac{\hat{\hat{\imath}}_{m_k}}{\hat{h}_k} = \frac{\bar{\kappa}(t)J_m \cdot s_k}{J_m \cdot \tilde{l}_k} = \bar{\kappa}(t)\frac{s_k}{\tilde{l}_k} \tag{S13}$$

where the subscript k indicates the coordinate axis direction. The symbols s and s̃ represent the areas of the primary and dual cells, respectively, while l and $\tilde{l}$ represent the edges of the primary and dual cells. The symbols ε, μ, σ, and κ correspond to the effective permittivity, permeability, conductivity, and magnetic loss, respectively.

To simplify the implementation of Eq. S7, it is essential to avoid the complex matrix operations inherent in classical Finite Integration Technique (FIT) algorithms. This can be achieved by employing a leapfrog scheme within the FDTD algorithm, specifically using the central difference method to discretize the time dimension in Eq. S7. For instance, assuming the function $f(t)$ is continuous at any time t, the derivative of $f(t)$ with respect to time t can be approximated as follows:

$$\frac{\partial f^n(n \cdot \Delta t)}{\partial t} \cong \frac{f^{n+\frac{1}{2}}\left(n \cdot \Delta t + \frac{\Delta t}{2}\right) - f^{n-\frac{1}{2}}\left(n \cdot \Delta t - \frac{\Delta t}{2}\right)}{\Delta t} \tag{S14}$$

where Δt represents the discrete time step. Substituting Eq. S9 and S14 into Eq. S7 yields the discrete recursive formula for Maxwell's equations in the time domain as follows:

$$\hat{\boldsymbol{e}}^{n+1} = \alpha_{ee}^{n} \cdot \hat{\boldsymbol{e}}^{n} + \beta_{eh}^{n+\frac{1}{2}} \cdot \left(\widetilde{\boldsymbol{C}} \cdot \widehat{\boldsymbol{h}}^{n+\frac{1}{2}} - \hat{\hat{\boldsymbol{\imath}}}_e^{n+\frac{1}{2}}\right) \tag{S15}$$

$$\widehat{\boldsymbol{h}}^{n+\frac{1}{2}} = \alpha_{hh}^{n-\frac{1}{2}} \cdot \widehat{\boldsymbol{h}}^{n-\frac{1}{2}} - \beta_{he}^{n} \cdot \left(\boldsymbol{C} \cdot \hat{\boldsymbol{e}}^{n} - \hat{\hat{\boldsymbol{\imath}}}_m^{n}\right) \tag{S16}$$

where n represents the discrete time step. The iterative coefficients α_{ee}, β_{eh}, α_{hh}, and β_{he} encompass the geometric parameters and material properties of each Yee grid cell, defined as follows:

$$\alpha_{ee}^{n} = \frac{[\bar{\varepsilon}(n \cdot \Delta t)/\Delta t - \bar{\sigma}(n \cdot \Delta t)/2]}{[\bar{\varepsilon}(n \cdot \Delta t)/\Delta t + \bar{\sigma}(n \cdot \Delta t)/2]} \tag{S17}$$

$$\beta_{eh}^{n+1/2} = \frac{(l_k/\tilde{s}_k)}{[\bar{\varepsilon}(n \cdot \Delta t + \Delta t/2)/\Delta t + \bar{\sigma}(n \cdot \Delta t + \Delta t/2)/2]} \tag{S18}$$

$$\alpha_{hh}^{n-1/2} = \frac{[\bar{\mu}(n \cdot \Delta t - \Delta t/2)/\Delta t - \bar{\kappa}(n \cdot \Delta t - \Delta t/2)/2]}{[\bar{\mu}(n \cdot \Delta t - \Delta t/2)/\Delta t + \bar{\kappa}(n \cdot \Delta t - \Delta t/2)/2]} \tag{S19}$$

$$\beta_{he}^{n} = \frac{(\tilde{l}_k/s_k)}{[\bar{\mu}(n \cdot \Delta t)/\Delta t + \bar{\kappa}(n \cdot \Delta t)/2]} \tag{S20}$$

where l_k and $\tilde{l}_k$ represent the side lengths of the primary and dual cells, respectively, while s_k and $\tilde{s}_k$ correspond to the areas of the primary and dual cells. Eq. S15 is the recursive formula for TDFIT. To simplify the implementation process, we express the discrete recursive form of the time-domain Maxwell's equations as follows:

$$\hat{e}_d\big|_{i,j,k}^{t+\Delta t} = \alpha_{ee}\big|_{i,j,k}^{t} \cdot \hat{e}_d\big|_{i,j,k}^{t} + \beta_{eh}\big|_{i,j,k}^{t+\Delta t/2} \cdot \left\{ \bar{\hat{h}}_{d_{prior}}\Big|_{\langle i,j,k\rangle_{d_{next}}+1}^{t+\Delta t/2} - \bar{\hat{h}}_{d_{next}}\Big|_{\langle i,j,k\rangle_{d_{prior}}+1}^{t+\Delta t/2} \right\} \quad \text{(S21)}$$

$$\hat{h}_d\Big|_{i,j,k}^{t+\Delta t/2} = \alpha_{hh}\big|_{i,j,k}^{t-\Delta t/2} \cdot \hat{h}_d\Big|_{i,j,k}^{t-\Delta t/2} + \beta_{he}\big|_{i,j,k}^{t} \cdot \left\{ \bar{\hat{e}}_{d_{next}}\Big|_{\langle i,j,k\rangle_{d_{prior}}+1}^{t} - \bar{\hat{e}}_{d_{prior}}\Big|_{\langle i,j,k\rangle_{d_{next}}+1}^{t} \right\} \quad \text{(S22)}$$

where $t = n \cdot \Delta t$, and the subscripts d, d_{prior}, and d_{next} follow the right-hand rule. $d = xyz$ denotes the coordinate axes. Specifically, the subscript d_{prior} represents the preceding spatial direction, and d_next represents the subsequent spatial direction. For example, if $d = x$, then $d_{prior} = z$ and $d_{next} = y$. Thus, the simplified terms on the right side of Eq. S21 and S22 can be expanded as follows:

$$\bar{\hat{e}}_y\Big|_{\langle i,j,k\rangle_z+1}^{t} = \hat{e}_y\Big|_{i,j,k+1}^{t} - \hat{e}_y\Big|_{i,j,k}^{t} \quad \text{(S23)}$$

$$\bar{\hat{h}}_z\Big|_{\langle i,j,k\rangle_y+1}^{t+\Delta t/2} = \hat{h}_z\Big|_{i,j+1,k}^{t+\Delta t/2} - \hat{h}_z\Big|_{i,j,k}^{t+\Delta t/2} \quad \text{(S24)}$$

Based on the definitions of iterative coefficients α_{ee}, β_{eh}, α_{hh}, and β_{he}, their specific values can be determined by considering the electromagnetic parameters ε, μ, σ, and σ_m, the time interval Δt, and the geometric parameters of the Yee grid. For perfect electric conductor (PEC) materials, the iterative coefficients are set as $\alpha_{ee} = 1$, $\beta_{eh} = 0$, $\alpha_{hh} = 1$, and $\beta_{he} = 0$:

$$\begin{cases} \hat{e}_d\big|_{i,j,k}^{t+\Delta t} = \hat{e}_d\big|_{i,j,k}^{t} \\ \hat{h}_d\Big|_{i,j,k}^{t+\Delta t/2} = \hat{h}_d\Big|_{i,j,k}^{t-\Delta t/2} \end{cases} \quad \text{(S25)}$$

Through the rigorous derivation process above, we find that TDFIT does not approximate the spatial dimension, effectively reducing the staircasing errors inherent in traditional FDTD methods. The concept of piecewise integration introduced in Eq. S22 bears significant similarity to the conformal techniques used in FDTD algorithms. In the mathematical formulation of TDFIT, we employ a central difference approximation only for the time dimension. This approach aims to circumvent the complex matrix operations inherent in classical FIT, thereby simplifying the implementation process and reducing computational complexity.

The application of the TDFIT not only effectively addresses dynamic electromagnetic problems but also opens new possibilities for real-time monitoring of complex biological systems. In practical applications, this method can be extended to simulate the electromagnetic properties of various complex media and time-varying environments, such as in medical

imaging, wireless communication, and metamaterial design. By further optimizing the algorithm and improving the utilization of computational resources, TDFIT is poised to become a standard tool for solving dynamic electromagnetic problems in the future.

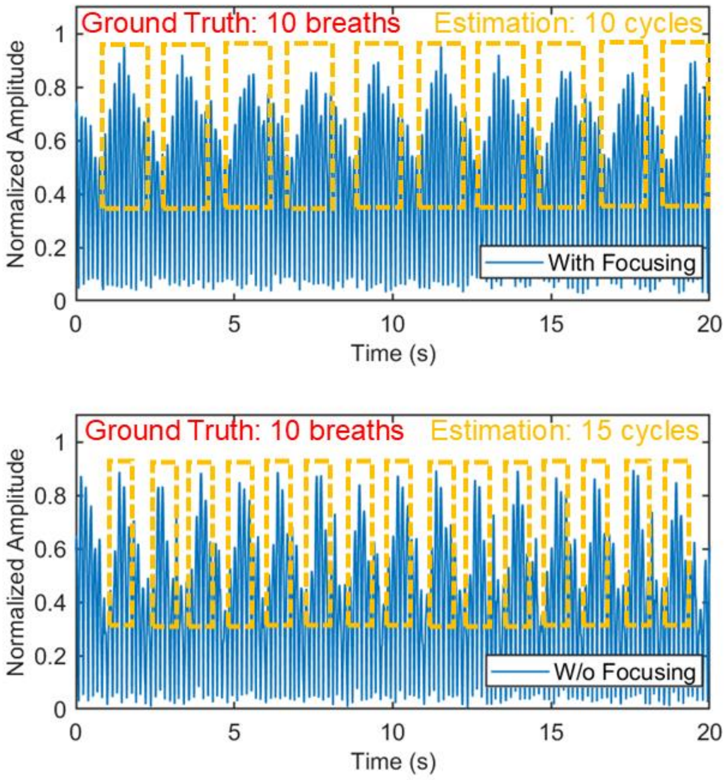

Fig S3 The time-domain human echo signals simulated with CST. (a) The human echo signal when there is a metasurface focusing the EM wave on the human chest. (b) The human echo signal when there is a metal surface mirroring the EM wave.

3 Near-Field Simulation of Human-Robust Vital-Sign Sensing

Fig S3 shows the simulated time-domain human echo signals. Since there is no ambient noise in the CST simulation, the periodicity of the human echo signals is induced by the motions of the human chest and arms. Specifically, the simulated human model in Fig S3(a) and (b) takes 10 breaths. As shown in Fig S3(a), there are 10 cycles in the human echo signal when this is a metasurface for EM wave focusing, indicating that the main component of the echo is the respiration signal. In contrast, as shown in Fig S3(b), there are 15 cycles in the human echo signal when the EM wave is not focused on the chest, which is not consistent with the periodic of respiratory movement. This is mainly due to the fact that in the absence of EM wave focusing, the EM signal is affected by both arm and chest movements. Therefore, the simulations demonstrate that the metasurface-based EM wave focusing can effectively mitigate the body noise induced by the other human body parts.

4 Experimental Results of Oblique Focusing

To achieve the focusing effect based on the GS algorithm, we first need to measure the actual wavefront amplitude and phase of the horn, as illustrated in Fig S4. Under a 45° oblique incidence, the wavefront exhibits excellent propagation characteristics, with clear structural visibility. Furthermore, we scale the measured amplitude and phase data into a 20×40 matrix format to facilitate subsequent iterative calculations using the GS algorithm. This matrix transformation not only simplifies the computational process but also enhances the accuracy and efficiency of numerical simulations.

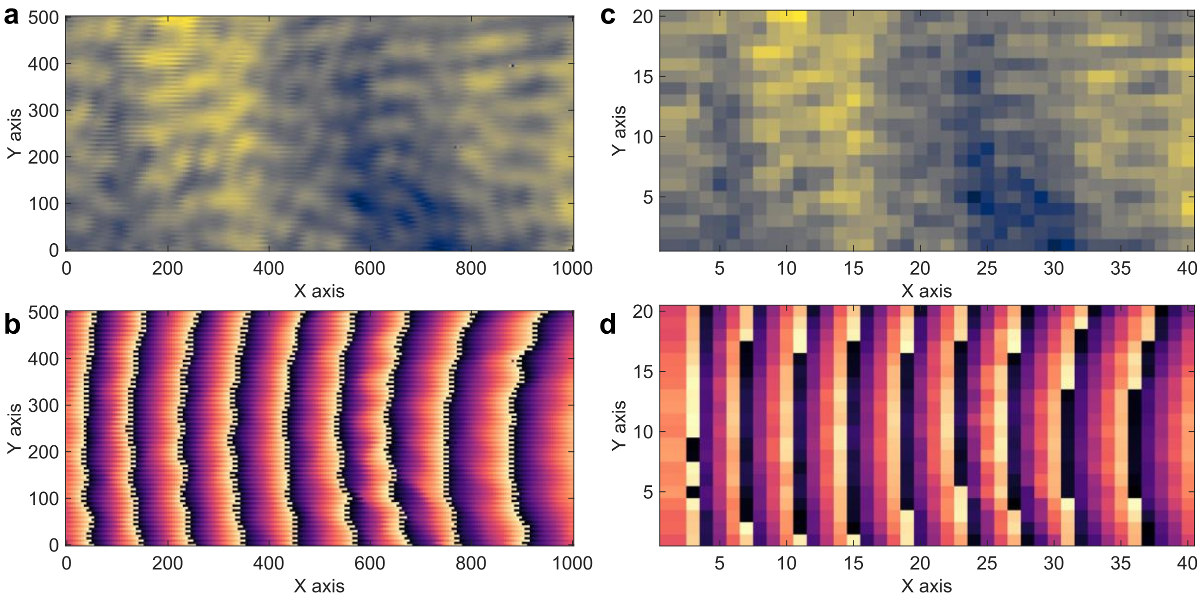

Fig S4 Measured wavefront of the horn. (a)-(b) Amplitude and phase of the measured wavefront of the horn. (c)-(d) Wavefront amplitude and phase scaled to a size of 20×40.

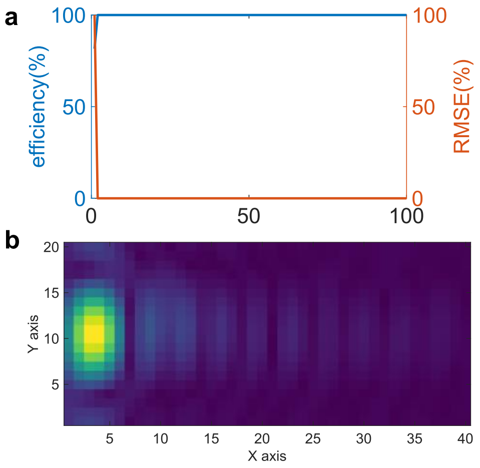

Fig S5 The computational process and results of the GS algorithm. (a) Iterative process for solving the full-phase holographic focusing. (b) Resulting focused electric field obtained using the GS algorithm.

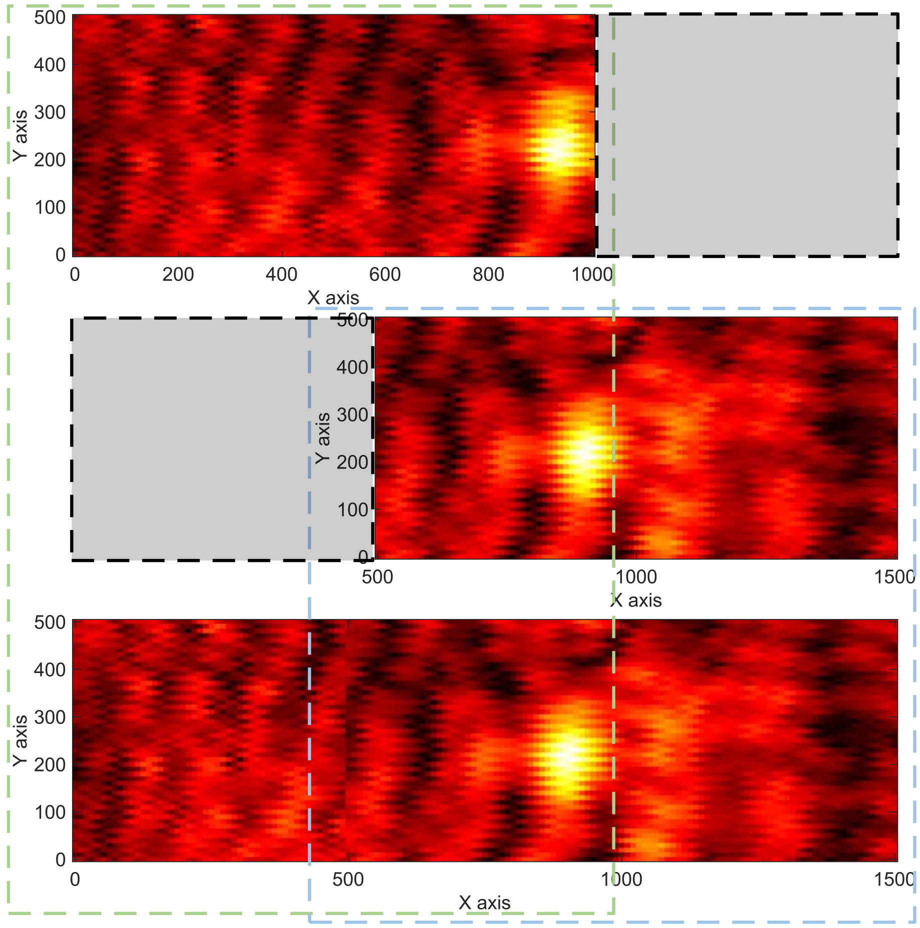

Fig S6 Measured near-field focusing results over a larger spatial range.

Subsequently, we input the obtained actual wavefront amplitude and phase data as initial values into the GS algorithm. It is noteworthy that the GS algorithm excels by iteratively optimizing to rapidly approximate the desired target field distribution. After a few iterations, as shown in Fig S5(a), we acquire a theoretical electric field distribution closely matching the target distribution, as depicted in Fig S5(b). In this instance, our target field distribution is designed such that only the central four points have an intensity of 1, with all other points having an intensity of 0. This design facilitates the achievement of precise focusing. Based on the final phase distribution matrix derived from the GS algorithm, we conducted further experiments to measure the near-field focusing results of the *BioMeta*, as shown in Fig S6. We not only present the near-field measurements within a 1000mm range but also supplement these results with measurements from the range of 1000-1500 mm. These data clearly demonstrate the realization of the desired near-field focusing at a frequency point of 3.9 GHz.

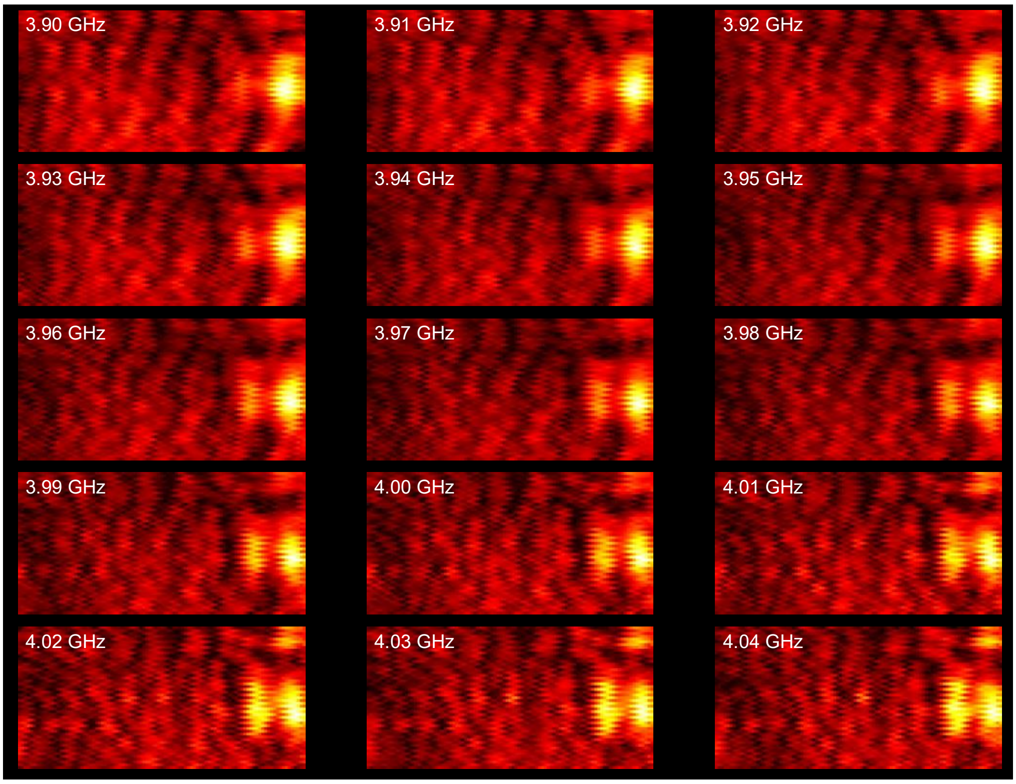

Fig S7 Experimentally measured near-field focusing results of the *BioMeta* at different frequency points.

Building on this foundation, we further expanded our research by demonstrating the measured results at different frequency points, as shown in Fig S7. The *BioMeta* consistently exhibits excellent focusing performance across multiple frequency points, highlighting the PB phase characteristics inherent in our designed metamaterial. These PB phase characteristics enable the metamaterial to achieve consistent and efficient wavefront control across various frequency conditions. It is important to note that the actual focal point is located on the left side of the images, and the horizontal coordinates in Fig S6 and S7 are reversed for better visualization. This adjustment aids in more intuitively displaying the correlation between experimental results and theoretical analysis, further validating the effectiveness and reliability of our design and implementation methods.

5 Comparison Experiments with and without EM Wave Focusing

To prove the effectiveness of the proposed *Green BioMeta* system, we employ a metal surface with the same size as the metasurface, and collect physiological signals of different individuals without EM wave focusing. The experimental setup for the scenarios with EM wave focusing and without EM wave focusing is shown in Fig S8(a) and (b), respectively. It is noted that all other configurations are the same in the comparison experiment, except for the difference in the

EM reflection medium. The other experimental setup is shown in Fig 4. Then, the electric field distributions of the metal surface and the metasurface are illustrated in Fig S8(c) and (d), respectively. It could be observed that the metasurface is capable of concentrating the energy of the EM wave at a specified location, while the electric field distribution of the EM wave reflected by the metal plate is random. As a result, there is less noise in the physiological signal extracted in the case of focusing than that in the case of unfocusing, as shown in Fig S8(e) and (f). Then, the electric filed distributions in Fig S8(c) and (d) are obtained when z and y are set to 1000 mm and 0 mm, respectively, as shown in Fig 5(b) and (c) of this paper. Fig S8(e) and (f) illustrate the extracted time-domain respiration signals under the scenarios with and without EM wave focusing, respectively. When EM waves are not focusing on the chest, there are obvious noise in the extracted signals (marked in yellow), leading to large estimation error of respiration rate. In contrast, the respiration signal with EM focusing (marked in red) has a similar periodicity to the benchmark signal and could be utilized for accurate respiration monitoring.

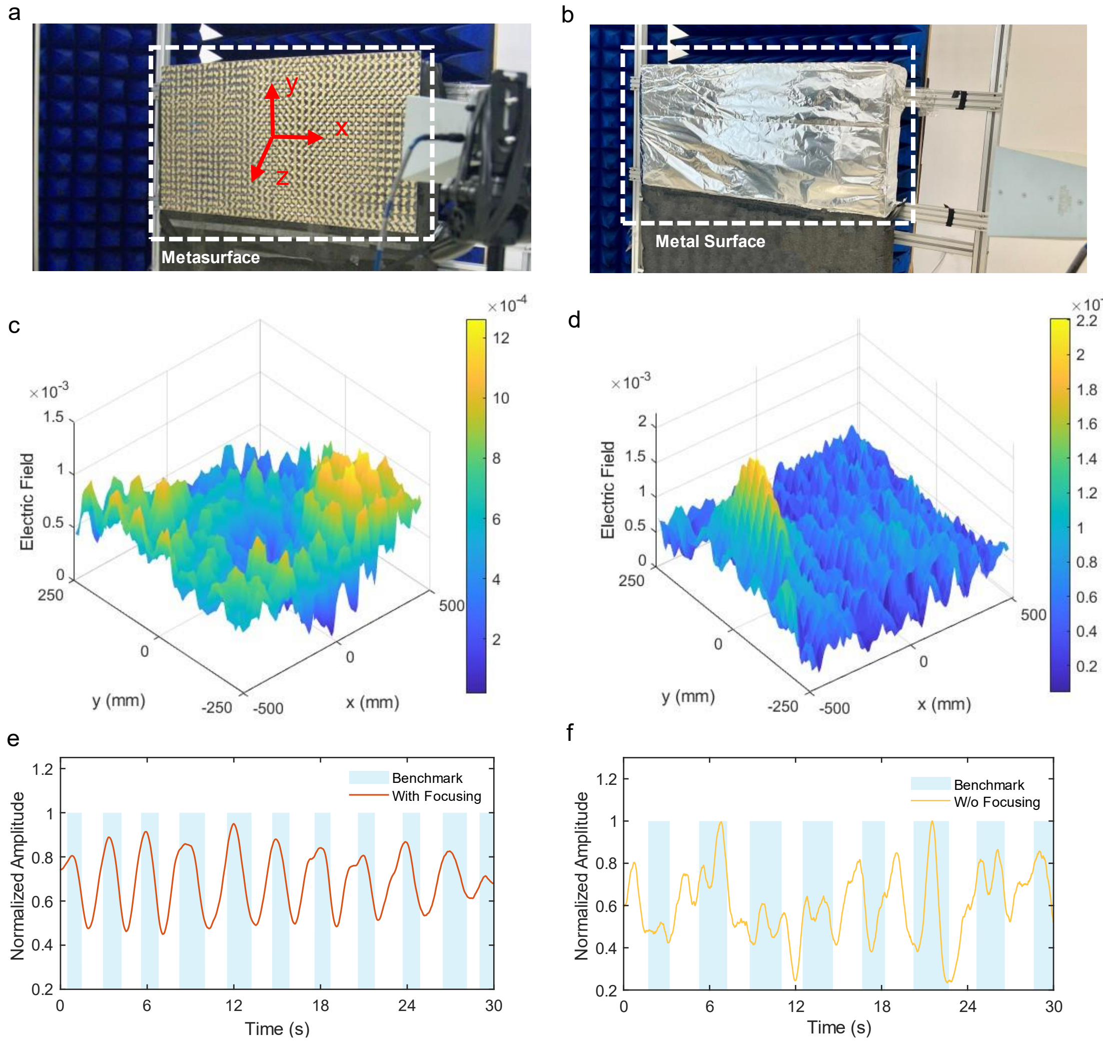

Fig S8 Experimental setup of with and without EM wave focusing. (a) The experimental scenario with the proposed *Green BioMeta* for EM wave manipulation. (b) The experimental scenario with a metal surface for EM wave reflection. (c)-(d) The electric filed distributions of the *Green BioMeta* and the metal suface, respectively. (e)-(f) The extracted time-domain respiration signals under the scenarios with and without EM wave focusing, respectively.

6 Estimation results of respiration rate for different individuals

Fig S9 illustrates the experimental results of respiration rate estimation for the two individuals *Bob* and *Alice*. The experimental setup is shown in Fig S9(a). Figure S9(b) and (c) provide the estimation results of *Bob* and *Alice*'s respiration rate within 60 s when the EM wave is reflected by a metal surface. Furthermore, by calculating the difference between the estimation and the benchmark, we can get the average estimation error with and without focusing, respectively, as shown in Fig S9(d). The low average error rate (AER) in this subfigure demonstrates the effectiveness of the proposed method. Specifically, it could be observed that an AER of 0.5 RPM has been achieved with the proposed system, while the AER under the scenario without focusing is 7.9 RPM.

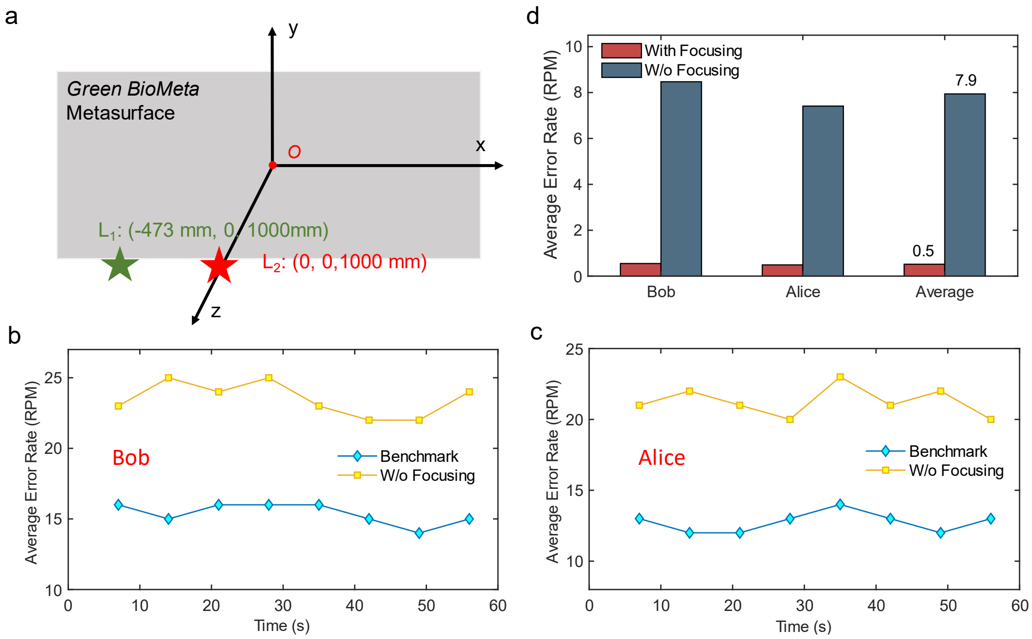

Fig S9 Experimental results of respiration rate estimation for different individuals. (a) The locations L_1 and L_2 of the two individuals *Bob* and *Alice*. (b)-(c) The estimated respiration rate of *Bob* and *Alice*, respectively, when the EM wave is reflected by a metal surface (W/o Focusing). (d) The histogram of average error for the two individuals in estimating respiration rate.
